# A Conspectus of the Different Forms of Consumption

**Published:** 1878-09

**Authors:** Roswell Park

**Affiliations:** Demonstrator of Anatomy, Woman’s Medical College; Surgeon to the South Side Dispensary


### Original Communications.

### A CONSPECTUS OF THE DIFFERENT FORMS OF CONSUMPTION, INTENDED AS AN AID TO DIFFERENTIAL DIAGNOSIS.

By Roswell Park, A. M., M. D.,

*Demonstrator of Anatomy, Woman's Medical College; Surgeon to the South Side Dispensary.*

(Based upon the labors of Niemeyer, Wagner, Rindfleisch, Ruehle and Virchow.)

The writer assumes that no apology is needed for any article calculated to throw any light upon the early recognition or differential diagnosis of the different forms of that dread malady—consumption. In the following contribution his sole endeavor has been to be *practical.* It would be impossible to present a complete picture of the disease within the limits allowed; much that is omitted must, therefore, be excused on that ground. The determination as to whether a case of phthisis before us is in a tuberculous condition; even further than this—to diagnose exactly the condition it may be in, is, as every one will recognize, of vast importance, especially as bearing on our prognosis and treatment.* That many advances have of late been made in this direction, we have reason to congratulate ourselves. Altogether too much carelessness exists in confounding the words "consumption" and "tuberculosis," "consolidation" and "deposit of tubercle," their indiscriminate use betraying some uncertainty in the minds of those who thus employ them.

So-called consumption, when advanced, can usually be diag-

* For an excellent summary of the benefits of change of climate in the different forms of phthisis, with advice as to choice of localities, see the address of Prof. Loomis before Section I. of the American Medical Association during its recent meeting at Buffalo; reported in the July number of this journal.

nosed by the merest tyro, but to recognize it in its formative stage, to differentiate it from other pulmonary complaints, to discern the tubercular element, are often matters requiring most careful scrutiny.

That patients live and die with phthisis without the formation of a particle of tubercle is an established fact; that others develop tubercle in the course of the complaint, and that in yet others the tuberculous process is the initiative step, are equally true.

Any assistance, therefore, to the separation and identification of each class of cases, is of practical importance. As a contribution in this direction, the writer submits the subjoined tabular views, hoping that they may prove as useful to others as they have been to himself.

But there are many points in the general appearance of a patient with incipient phthisis which may be regarded as aiding in the diagnosis or showing a condition of system favorable to its development. At the risk of being prolix, the writer desires to mention the following: slender bones, thin skin, delicate hue of cheeks, large eyes, bluish sclerotic, long eyelids, pale face which blushes readily, little subcutaneous fat, thin, slender hands with incurvated nails (the clubbed fingers are not distinctive of phthisis as many suppose; they are noticed in several chronic complaints), sharply defined red line at the edge of the gums opposite the canines and incisors; poorly-shaped muscles—especially those of the neck which allow the thorax to sink and thus cause the neck to appear long, small antero-posterior diameter of thorax, wide interspaces, acute angle of junction of ribs to sternum—causing the chest to seem long, flat and narrow, shoulders tipped forward and inner angles of scapulæ tipped up like wings; abdominal character of respiration, spots of pityriasis (tinea) versicolor or lentigo hepatica on back and chest; rise of temperature (38.5 C.) with flushed cheeks and hot hands *at evening*, gradual emaciation, wandering pains in chest and shoulders, etc.

Among the physical signs may be mentioned—special excitability of the heart in anæmic persons, continuous acceleration of the pulse without evidence (except by the thermometer) of increased temperature, systolic murmur in the subclavian, becoming louder or heard only during expiration (a sign of adhesion of pleural surfaces at apices). The pulse is not only rapid, but soft and empty, and even when there is fever it has not the usual sthenic characteristics. Enlargement of glands may cause venous compression with dilatation of veins of neck and face, or even cyanosis. Depression of supra and infra-clavicular spaces does not always *of itself* indicate any variety of phthisis; it is due to induration or shrinking of the apex of the lung (from pneumonic-cirrhotic—processes) and may predispose. But when it appears by itself without other signs to indicate phthisis, it more often indicates a partial cure of the nutritive changes which of themselves predispose to that disease. A feebleness of respiration in the same locality and with similar accompaniments is of similar import. So also is a wide extension of cardiac shock and dislocation outward of apex beat. These latter signs, therefore, are by no means pathognomonic of phthisis unless accompanied by other signs, as above.

Caution is required if one would not be misled by too great reliance on physical signs. Puerile respiration and increased vocal resonance in right infra-clavicular and supra-spinous regions, often alluded to as signs of incipient phthisis, are met with in healthy lungs, and should not be regarded as suspicious sounds unless accompanied as above. So, too, with regard to fever; a fever which has no obvious cause, and which persists, points to phthisis; if there be absence of fever and suspicious physical signs, even though there be emaciation and cough (often caused by tracheal catarrh or chronic tonsilitis), the fear of phthisis may be dismissed.

Anæmia, combined with dyspepsia and hysteria, may simulate phthisis; here the thermometer should decide. Chronic bronchial catarrh may cause needless alarm; but this need not be unless it shall have attacked the apices; its usual manifestations are confined to the lower lobes, and as improvement occurs it takes place from above downward.

Phthisis may be strongly suspected when a patient with a cough has or has had an uncontrollable diarrhœa, or non-specific aphonia or when fever is present without other cause.

Without going at all deeply into the consideration of the ætiology of consumption, certain factors in which are mentioned in the tabular statements, a few either of the more prominent points, or of those too often overlooked, will bear mention in this connection; consideration of these points not infrequently influencing our diagnosis.

A sudden chilling of the stomach or of the skin may be sufficient to set up catarrhal or pneumonic processes whose termination we cannot forsee. Children fed on pap instead of being nourished at the breast, are more apt to react poorly against such disease. Diseases such as syphilis, typhoid, diabetes, chlorosis, etc, predispose, inasmuch as they cause malassimilation. Puny, badly nourished subjects, are more susceptible, because among such, inflammatory nutritive disorders show greater tendency to cell formation and subsequent caseous degeneration. Great alterations of temperature and a high degree of moisture each predispose, as do previous and frequent catarrhs.

Lack of expansibility of chest apices owing to change in the cartilages of the first ribs, congenital or occurring in early youth (e. g. ossification or shortening), faulty carriage of body, diminished respiratory capacity, due to feebleness of respiratory muscles, are all prominent factors. Bronchial catarrh and usually associated disease of the bronchial glands attending measles, especially when epidemic, and the frequent general glandular troubles also attending it, make it a frequent cause. The connection between phthisis and pertussis is, for the same reason, equally manifest. That between phthisis and the later stage of diabetes mellitus is more obscure.

Hæmorrhage, as such, does not predispose except as its resulting clots fail to be resolved and absorbed; clots or fragments of clots remaining may be regarded with considerable concern.

There is no evidence that the milk of consumptives can excite the disease.

On the other hand, malaria and valvular heart disease are said to confer some immunity. While the phthisical lung is the more vascular, emphysematous lungs are more dry and bloodless and rarely inflame; but when once inflamed they degenerate rapidly.

With regard to the cough, almost always a distressing feature, it may be regarded as the expression of (*a.*) the extent of implication of the bronchial mucous membrane; (*b.*) the extent of implication of the lymphatic (bronchial) glands; (*c.*) nervous reflex excitability; (*d.*) the proximity of the lesion to the larynx.

Table I. gives, side by side, the features of the three different forms of inflammatory phthisis uncomplicated by tubercle; the site of the primary lesions being respectively in the *interstitial connective tissue*, the *acini* or *alveoli* and the *bronchioles*. The second class of cases can usually be more easily differentiated from the first than from the third; while in many cases there are such a variety of pathological processes taking place consentaneously that it may be impossible to assign the patient a place in any one class.

But that cases are frequently met with where this classification can be made, I trust no one will deny. It is taken for granted that the tubercular element has been excluded (though perhaps this is not essential); this is done chiefly by aid of the thermometer (vide Table II.) This table is founded principally upon the monograph of Ruehle. (Ziemssen's Cyclop).

### Table I.

### *Uncomplicated Inflammatory Phthisis.*

| Simple Chronic Apicial Pneumonia. | Lobular Disease Characterized by Foci:—Broncho Pneumonia. | Apicial Catarrh. |
|---|---|---|
| Changes consist of simple, chronic, indurative inflammation. | Changes consist of formation of clusters of nodular foci which excite inflammation in surrounding parts and coalesce with the inflammatory products thus formed. The whole mass then becomes caseous, and breaking down forms ulcers, and so the destructive processes advance; or they may be arrested by the recurrence of simple inflammation with resulting induration. | Beginning in the bronchi, producing inflammatory infiltration and swelling of the entire thickness of their walls, with caseation and dilatation. Degeneration then takes place, pneumonic processes are set up by extension into the parenchyma, and the inflammatory products either necrose or undergo induration resulting in limitation and cure. |
| The most important physical sign is dullness in the supra, and infra-clavicular and supra, and infra-spinous fossæ. The more intense and extended this dullness, the greater the probability that this is the form of the disease. | Slight dulness, with sunken supra-clavicular fossæ; apices of lungs lowered. (Best ascertained by percussion in front, with patient's mouth open.) | First shades of dulness develop very gradually in apices, and sometimes a little further down. |
| Later the altered shape of affected side and impeded respiration show that shrinking has supervened. This sinking above and below the clavicle and the flattening of the whole upper part of the thorax indicate chronic pneumonia. | Narrowed chest, with imperfect and unequal expansion. | |
| | Signs of infiltration predominate over those of bronchitis. | Signs of bronchitis predominate over those of infiltration. |

| | | |
|---|---|---|
| Respiratory murmur changes gradually from a puerile respiration to a loud, harsh, bronchial sound, heard both during inspiration and expiration. | Expiratory sound is usually bronchial; and in many cases the inspiratory sound has an uninterrupted, vesicular character for an appreciable time before the sound becomes bronchial. | |
| Crepitant râles may be heard at times; if present, they are rather sibilant in character. If any catarrh be present, these may be abundant. | Crackling râles are scanty at the outset; but when softening takes place, they are abundant, and mixed with a sibilant, bubbling sound. | Non-sibilant crackling râles are heard from the outset, at least on deep inspiration and coughing. Later the râles may become sibilant. |
| The local signs extend very slowly, or as gradually become confined and fixed. | Course more rapid. | Same. |
| If bronchiectases are produced by the cirrhosis and retraction of the connective tissue, signs of cavities of corresponding size will be found. | As the disease advances (*vid. supra*) auscultatory and other signs of cavities are discerned; *e. g.*, tympanitic and "cracked-pot" resonance. | Same. |
| Cough is usually of moderate severity, or may be absent. | Dry cough from the outset, with scanty expectoration. | Cough, with moderate expectoration, one of the first symptoms. |
| Expectoration scanty; but in case there be inter-current bronchial catarrh, it is more abundant, catarrhal in character and muco-purulent. | When expectoration is a feature, the sputa are gelatinous, of reddish tint, and contain elastic fibers early in the disease. | Expectoration accompanies the cough; sputa frequently contain streaks of blood. |
| Sputa contain no blood. | Later the sputa are more solid and globular, and contain more or less blood. | Sputa contain also yellowish-white, non-aerated streaks, and much amorphous matter, together with small detached particles which sink in water. |
| Hæmoptysis rarely or never. | Hæmoptysis may cause the first alarm. | Hæmoptysis not as likely to occur. |
| Pains—supposed often to be rheumatic—frequently occur on affected side. | More or less pain. | More or less pain. |

| | | |
|---|---|---|
| Usnally no complications. | Derangement of appetite and digestion frequent. Functions of larynx and intestines rarely undisturbed. | Laryngeal and intestinal complications make their appearance late. |
| Fever—at evening—absent or inconsiderable. | Fever — at evening — is present, with night sweats. | Fever inconsiderable until the disease is advanced. |
| Emaciation — at first only slight — increases very slowly or disappears. | Emaciation one of the earliest and most prominent symptoms; advances *pari passu* with the fever and complication. | Emaciation and loss of strength, with constitutional symptoms, are among the first suspicious signs noticed. |
| Heredity plays an unimportant part. | Heredity plays an important part, as do rapid growth, poor development, and such diseases as typhoid, morbilli, pertussis, etc. | Heredity plays a conspicuous part, especially in "scrofulous" patients, or those who have frequent attacks of catarrh. |
| Local irritating causes are a much more important factor. | Local irritating causes are a much less important factor. | Same. |

Thus much for the cases of purely inflammatory phthisis. The frequent complication of such cases by the neoplastic element, *tubercle*, makes possible yet another grouping between, on the one hand, those cases included in Table I, and, on the other, certain cases where the appearance of miliary tubercle is, so far as can be determined, the first pulmonary lesion. Between these two strongly contrasted forms occurs one where tubercle is found in products of previous inflammatory change.

Our knowledge of tubercle is yet far from exact, and the dividing line between it and scrofulous and other affections not clearly drawn. But the writer may be allowed to define the position taken in preparing this paper. With regard to scrofula, Ruehle's views seem so just and well-founded, that they are given almost verbatim.

By scrofula is meant—not any particular feebleness or sickliness of body, but rather a tendency to certain forms of disease, e. g., inflammations of mucous membranes and skin, whose products are abnormal in character, and which being carried to the lymphatics excite in them inflammation or hyperplasia. These inflammations are characterized by abundant cell proliferation. According to Rindfleisch, one fundamental characteristic of scrofulous affections is the accompanying disproportion between the volume of the blood and the weight of the body.

Scrofulous, as well as other inflammations lead often to the formation of cheesy masses. Tnese are described by Wagner as being results of inflammation where the purulent or fibrino-purulent exudation, or the desquammated epithelium have, in consequence of anæmia, lost so much liquid that there results a dry, grayish or yellowish mass, firmly imbedded in the tissues. Even tubercle itself, as well as old cancerous nodules, hæmorrhagic infarctions, incapsulated collections of pus, etc., may undergo this change as well as fatty metamorphosis. There is no *yellow tubercle* as formerly spoken of; when such is found, it is the product of the cheesy atrophy just mentioned.

Now these cheesy infiltrations and suppurations of mucous membranes, by some unknown means, elaborate a poison which, when re-absorbed, produces tubercle. The source of tubercular infection is, therefore, thus furnished by the patient himself. (Rindfleisch.)

Probably the best idea or definition of tubercle may be gathered from the description of Wagner, who considers it an infiltrated, multiple, usually miliary, non-vascular neo-plasm, consisting especially of nuclei of varying sizes, indifferent and giant cells—all imbedded in a reticular tissue, and which constantly tends to pass into cheesy atrophy or softening. The basis for its development—the cheesy focus—is most frequent in connective tissue, previously irritated ("scrofulous") glands, bones, testicles, etc. This cheesy focus may be incompletely encapsuled, the capsule being only a relative and considerable but not absolute obstacle to resorption. Tubercle probably originates from (fixed) connective tissue corpuscles, and the endothelium connected therewith; it grows by division of the nuclei and extends along connective tissue, lymphatics and blood-vessels.

Tuberculosis has its analogies to miliary carcinosis; is probably transmissible, like glanders, syphilis, etc., and is not usually found with other infectious diseases.

With regard to the prognosis of cases of consumption thus complicated, resorption of tubercle, accompanied usually by fatty atrophy, takes place very rarely indeed; calcification, softening and liquefaction (ulceration), much more frequently. But there is never a chance that the lung can return to its normal condition. The best that can be hoped for is that the lesions already existing may be rendered innocuous; this happens by shrinkage of the infiltration and formation of blood-vessels which do not penetrate deeply, but supply constant though scanty nourishment. (Rindfleisch, Wagner.) Of course this applies only to cases referred to in the second column of Table II.

These remarks will suffice to explain more fully the following table. If there be any one point in it upon which sufficient stress has not been laid, it is that in reference to the constant use of the thermometer as an aid to diagnosis. The writer deems it possible by its aid, and by the other signs given in the same table, to group four-fifths, at least, of cases of consumption in their proper place; and of those coming under the heading of the first column probably one half can, by the use of Table I, be again classified.

### Table II.

*Non-Tubercular Compared with Tubercular Phthisis.*

| Inflammatory. | Tubercular. | Acute Miliary Tuberculosis. |
|---|---|---|
| *Purely and Directly the Result of Inflammatory processes.* May be either one or a complication of all the forms mentioned in Table I. | *Adventitious Deposit of Tubercle (Miliary) following Caseous Metamorphosis of Products of Inflammation.* Always secondary to the inflammatory variety. | *Supervention or Formation of Miliary Tubercle during Apparent Good Health.* Much resembles an acute infectious disease. |
| Ætiology, etc. | Ætiology, etc. | Ætiology, etc. |
| Exciting causes: anything which produces congestion and bronchial catarrh. (*Vid. supra.*) | A sort of middle ground between the forms on either side.<br>Occurs in connection with pre-existing lesions of the apices.<br>The greatest danger for consumptives is that they may become tuberculous. Incapsulation of caseous masses affords some protection against this. | But one cause is known; the absorption of caseous matter. Most cases present some antecedent lesion of some organ; but often not recognized.<br>Persons predisposed to inflammatory action suffer from acute and primary tuberculosis with greater relative frequency. |
| Pneumonia, catarrhal or croupous, when it has attacked the apex. | Next after the caseous products of pneumonia in liability to tubercular infection come the exudates of pleurisy and pericarditis; caseous, lymphatic and bronchial glands, etc. | Where measles, etc., or "scrofulous" affections have caused enlarged lymphatics, bronchial glands, etc., or where there are caries of bone, caseous testicle, prostate or bladder, remains of inspissated abcesses, remains of serous inflammations or ulcerations, or the like, there may be said to be cause for development of miliary tubercle. When it assumes the form of an |
| Croup, cerebral irritation, or moist eruptions, may be followed, after puberty, by bronchial hæmorrhage and inflammatory lung disorder. | | |

| | | |
|---|---|---|
| | | acute disease, these foci may be considered as having for some time remained non-infective. |
| Pertussis, morbilli, scarlatina, etc., may lead to this form directly. | Pertussis, morbilli, scarlatina, etc., may lead to this form by producing a "scrofulous" condition and glandular tuberculosis, thus supplying foci for subsequent general infection. | Same. |
| PATHOLOGY. | PATHOLOGY. | PATHOLOGY. |
| Is the result of inflammatory processes, and is a chronic disease with intercurrent simple forms of inflammation, which may heal by cicatrization. | Is the result of a neoplasm; is more or less chronic, and is a resorption disease. | Is the result of a neoplasm; is rapidly acute, and is a resorption disease. |
| Consists in suppuration or caseous degeneration of lobular or lobar infiltration; the extension (with exceptions) of a chronic catarrh and copious secretion of young cells into the bronchioles and finally into the air vessicles. | Follows caseous infiltration or degeneration as a sequel to chronic catarrhal or croupous inflammation, tubercles being deposited in the metamorphosed pneumonic product. | The focus of attraction for the acute production of tubercle in the lungs is usually a pre-existing chronic tuberculosis of those organs; when miliary tubercle forms the only lesion we have to do with the acute infectious disease. |
| | Is regarded by some as a combination of inflammation and tuberculosis. | There is no chronic miliary tuberculosis in the *old* sense of the word. |
| | Plays a subordinate rôle, inasmuch as tubercle is an accidental, secondary product. | |
| | The number, size and distribution of of miliary tubercles do not necessarily | Same. |

| | | |
|---|---|---|
| | afford an expression of the constitutional disturbance. | |
| | When upon phthsis tubercle supervenes, it usually first develops in the mucous membrane of the bronchi, trachea or larynx. The most frequent site is the point where the bronchiole connects with the acinus. Thence it spreads in all directions. | In the invasion of miliary tuberculosis three stages are often distinguishable: (*a.*) Local scrofulo-tubercular primary infection; (*b.*) tuberculosis of lymphatic glands; (*c.*) general infection. |
| When the branches of the pulmonary artery become obliterated, the bronchial arteries enlarge and conduct the supply of blood to the lungs. The intercostal arteries also enlarge and advance through the pleuritic exudate; so that the affected lung really receives more blood than the sound one. Part of this blood is discharged into the intercostal veins; this impedes the discharge of the cutaneous veins into the intercostal so that the former enlarge; and hence the blue network of veins often noticed on the chest wall. | Same. | Time is not afforded for these changes. |
| Cavities are enlarged by the same processes which assisted in their formation. | Cavities rarely enlarge by caseous degeneration of the tubercular infiltration, but by a diphtheritic infiltration with subsequent decay. | Cavities are not formed. |
| Often accompanied by laryngeal and intestinal diseases, as well as by fatty or amy- | Often combined with laryngeal disease, ulcer of the bowel, intestinal or cerebral | May be complicated by intestinal as well as cerebral or meningeal tubercle. |

| | | |
|---|---|---|
| loid changes in the liver; with the access of the former (fatty change) occurs imperfect assimilation of food and emaciation; with the access of the latter the pulmonary symptoms often seem to recede. | tubercle, and fatty or amyloid liver and kidneys. | |
| In protracted cases the volume of the blood is diminshed and the heart flabby. | Same. | Cases cannot be considered protracted; nevertheless the same change may occur. |
| In recent cases the flow of the right heart is impeded, and it is therefore dilated and hypertrophied. | | |
| Never appears before first dentition. | Same. | May occur in early infancy. |
| MORBID ANATOMY. | MORBID ANATOMY. | MORBID ANATOMY. |
| The anatomical appearances are so varied and inconstant, and so familiar, that space cannot be afforded to describe them. Suffice it to say, that among all the appearances of infiltration, dilatation, caseation, ulceration and necrosis, *no* tubercles are found. | The tubercle of chronic tuberculosis is never uniformly disseminated; the gray tubercle proper being found along with the yellow caseous masses (the so-called "yellow tubercle") showing the deposit to have gradually taken place. The abdominal viscera are less often covered with tubercles. | Lungs are congested, œdematous, emphysematous at edges, and present in the vast majority of cases an uniformly studded aspect of miliary tubercles, gray, translucent and fresh. Pleuræ the same. The peritoneum, liver, kidneys and spleen are usually covered with the same. Also, in young subjects, the parts about the base of the brain. The smallest miliary tubercles, being invisible to the unaided eye, are often overlooked in the glands and viscera. |
| After death the skin is remarkably white and covered with scales of pityriasis (tinea) tabescentium. Feet and limbs are often | Same. | The corpse usually resembles that of one dead from acute febrile disease, decomposing rapidly. The blood is dark and liquid and settles to dependent parts, causing pul- |

| | | |
|---|---|---|
| œdematous, and crural veins plugged with thrombi. | | monary hypostases and suggillations The muscles are friable. The spleen soft and enlarged. |
| A catarrhal ulcer of the intestine should not be mistaken for tubercular. | Many so-called tuberculous ulcers of the intestines are of a lardaceous or catarrhal character. | Intestinal ulcers very rarely form. |
| | The center of the tubercle usually degenerates first. | Same. |
| Caseous bronchial glands infrequent. | Caseous bronchial glands frequent. | Same. |
| | | As there has been more or less bronchial or tracheal catarrh during life, evidences thereof are found after death. |
| SYMPTOMATOLOGY AND SEMEIOLOGY. | SYMPTOMATOLOGY AND SEMEIOLOGY. | SYMPTOMATOLOGY AND SEMEIOLOGY. |
| Has a period of percussory catarrh, with protracted cough and expectoration, of variable duration; this need not necessarily commence in the alveoli and bronchioles, or connective tissue, but may proceed downward from the trachea. | Is necessarily preceded by some such condition as that just described. | At outset repeated rigors, without complete intermission, with rapid pulse increasing respiratory rate, and severe constitutional disturbance. |
| Fever and wasting deferred. | Pallor, fever, emaciation and night-sweats usually appear early with cough and expectoration. | Very much resembles typhus. Anorexia a notable symptom ; mouth often dry, at times very moist. Rapidly increasing prostration with aggravated cough and dyspnœa. Extremities cool. Slight cyanosis. Soon colliquative sweats, pulse more |

| | | |
|---|---|---|
| | | rapid, emaciation from day to day, sensorium deranged and semi-coma. |
| | Spleen may be slightly enlarged. | Spleen is usually enlarged. |
| Physical signs are enough to explain other symptoms. | Same. | Are not enough. |
| Well defined, dark yellow streaks in sputa, showing that the catarrh has reached the finer bronchi; a bad sign.<br>Infiltration and caseous degeneration may occur without cough or expectoration. Should intestinal inflammation complicate such cases it may exercise a derivative influence on the bronchi. | | Condition of sputa crude and dependent upon the amount and extent of bronchial catarrh; they possess no characteristic appearances. |
| Voice and cough rarely hoarse. | A hoarse or inaudible cough or a distressing laryngitis may occur with the advent of tubercular complication, and are pathognomonic and unfavorable signs. | Cough may be distressing, but hoarseness is rare. |
| The discovery of elastic fibres in the sputa is a positive sign. | The same holds good here; and when the sputa of a persistent cough, accompanied by fever, for a long time retain the crude character of the mucous sputa of acute bronchitis, suspect development of tubercle, since the existence of tubercle in bronchial mucous membrane is generally attended with distressing cough and scanty sputa. | The cough of this variety may be thus accounted for. |

| | | |
|---|---|---|
| The appearance of blood *in* muco-purulent sputa, whereby they acquire a yellowish-red color, indicates a chronic pneumonia, and shows that catarrh has extended to the air vesicles.<br>Rounded, nummulated sputa, which sink slowly to the bottom, and do not coalesce, are sure indications of a *cavity*, but not necessarily of tuberculosis. | | |
| The hectic is more of a *remittent* or *intermittent* than of a continued type; with a range of, say, 1.1° C. between evening and morning temperature; the evening elevation being a constant feature.<br>The fever may present all possible variations in the same individual. A sudden accession may be regarded as an indication of some fresh inflammatory process; *e. g.*, pleuritis, pneumonia.<br>(In children the invasion of the air vesicles by an acute catarrh is accompanied by increased temperature and accelerated pulse.) | The hectic is of a *continued* type: temperature always above normal, but not much higher in the evening than in the morning; *i. e.*, the remissions *not* well marked; moreover it resists treatment. | Temperature is of the *remittent* type; but not infrequently the maximum occurs during the morning. It rarely rises very high at any time, and towards the end the mean temperature may decline.<br>Pulse becomes smaller, and yet more rapid, while the respiration rate increases; œdema of lungs from insufficient filling of right heart, and finally palsy of bronchi and suffocative effusion; the violence of the fever, malignancy of the disease, dyspnœa and rapid collapse, affording data for differential diagnosis. |
| With marked evening rise of temperature, the rate of respiration does not correspondingly accelerate; hardly ever more than six or eight breaths per minute. | Same with regard to period of maximum temperature; only the temperature range does not show such sharp curves. | Temperature very seldom reaches 40° C., and is out of all proportion to the rapidity of the pulse. |
| Patients may run through the whole | Diarrhœa, especially in those of costive | Little or no meteorism or ileo-cæca |

| | | |
|---|---|---|
| course without diarrhœa or evidences of meningeal complication. | habit; signs of implication of meninges, especially in younger subjects; also hoarseness and aphonia. | tenderness. Stools are indefinite in color and consistence. |
| Profuse sweating, usually at night. | Same; with sudamina. | Profuse sweating at no particular time; sudamina frequently. |
| Pain in shoulders and chest more frequent. | Pain in shoulders and chest less frequent. | Little or no pain in shoulders or chest. |
| A white coat of vegetable spores and filaments often found on tongue. | Same. | |
| Painful decubitus in last stage; with œdema of feet. | Same. | Patient usually succumbs in from two to six weeks. |
| May terminate with hæmorrhage. | Same. | The disease may be complicated with tubercular meningeal trouble, and then be even more rapidly fatal. |
| PHYSICAL SIGNS. | PHYSICAL SIGNS. | PHYSICAL SIGNS. |
| With patient's mouth open it is not difficult to mark out, in front, the upper limits of the lungs—an important matter. (*Vid.* Table I). | A feeble respiratory murmur where the percussion note is normal or abnormally resonant is indicative either of tubercular trouble or lobular pneumonia. | Any previous dullness or history of pulmonary disorder is of great significance. |
| Rapid respiration and even dyspnœa *with* dullness. | Rapid respiration and even dyspnœa *without* dullness. | Same. Respirations may run up to 50 or 60 in the minute, but without correspondingly increased exertion characteristic of true dyspnœa. |
| Physical examination usually reveals enough to account for other symptoms. | Physical examination usually fails to reveal enough to account for *all* the symptoms. | Same. |
| Solidification of lung usually extensive. | Even after consecutive pneumonia, with bronchial breathing and ringing râles, solidification *not* extensive. | No solidification or infiltration. |

| | | |
|---|---|---|
| When area of dullness accords with the other signs it is a comparatively favorable feature. | Tubercle *of itself* never gives rise to sufficient consolidation to cause dullness. | Seldom any perceptible abnormality of percussion note, or but very slight change, as compared with the other side. |
| The presence of lobular infiltration may, in some cases, cause a hollow or tympanitic note. | The presence of miliary tubercle may, in exceptional cases, produce the same percussion sound. | *Vid. supra.* |
| 'Cracked-pot' resonance over a cavity with thin walls. | Same. | |
| Fremitus is intensified over cavities connecting with bronchi and containing air. (Of little value as a sign.) | Same; fremitus may also be exaggerated by lobular infiltration and by tuberculosis. | |
| Bronchial respiration, broncophony, and sonorous râles are heard after extensive induration. | Same. | Sometimes a soft friction murmur, caused by tubercular deposit on pleural surfaces, is heard. (Juergensen.) |
| In the first stage, feeble, harsh or puerile respiratory sounds are heard, with all the signs of catarrh at apices. (*Vid.* Table I.) | | The different signs of catarrh, accentuated inspiration with audible expiration, whistling, sonorous bronchi over whole lung; and later râles, heard first in dependent regions. |

125 State St., Chicago.

